# The Curvilinear Relationship between Age and Emotional Aperture: The Moderating Role of Agreeableness

**DOI:** 10.3389/fpsyg.2017.01200

**Published:** 2017-07-18

**Authors:** Anna Faber, Frank Walter

**Affiliations:** Department of Organization and Human Resource Management, Justus Liebig University Giessen Giessen, Germany

**Keywords:** age, emotional aperture, emotion recognition, group emotions, agreeableness

## Abstract

The capability to correctly recognize collective emotion expressions [i.e., emotional aperture (EA)] is crucial for effective social and work-related interactions. Yet, little remains known about the antecedents of this ability. The present study therefore aims to shed new light onto key aspects that may promote or diminish an individual’s EA. We examine the role of age for this ability in an online sample of 181 participants (with an age range of 18–72 years, located in Germany), and we investigate agreeableness as a key contingency factor. Among individuals with lower agreeableness, on the one hand, our results indicate a curvilinear relationship between age and EA, such that EA remains at a relatively high level until these individuals’ middle adulthood (with a slight increase until their late 30s) and declines afterward. Individuals with higher agreeableness, on the other hand, exhibit relatively high EA irrespective of their age. Together, these findings offer new insights for the emerging literature on EA, illustrating that specific demographic and personality characteristics may jointly shape such collective emotion recognition.

## Introduction

Correctly recognizing and deciphering others’ emotion expressions is an important interpersonal skill that critically shapes social functioning (Carstensen et al., 1997; Keltner and Kring, 1998). With emotional cues providing information about individuals’ feelings, opinions, and intentions, the ability to assess and interpret others’ emotions helps to create effective and harmonious social interactions and, thus, is vital for success in interpersonal communication (e.g., Byron, 2007; Isaacowitz et al., 2007).

Recent theory suggests that it is often important to grasp not only other *individuals’* emotion displays, but to simultaneously assess the emotions expressed by a group of others (Sanchez-Burks and Huy, 2009). Such “emotional aperture” (EA) reflects the ability to recognize a group’s overall emotional composition by focusing on the global picture of diverse emotion expressions within a collective (Sanchez-Burks and Huy, 2009; see also Navon, 1977). With group and team structures permeating many modern organizations (Kozlowski and Ilgen, 2006) and groups’ emotional setup shaping important processes and outcomes (George, 1996; Barsade et al., 2000; Walter and Bruch, 2008), the capacity to accurately decipher collective emotionality is likely to be critical for employees’ functioning. Accordingly, research has illustrated EA as a unique capability (distinct from individual emotion recognition) that relates with important behaviors and outcomes in organizations (e.g., leaders’ transformational behavior toward their followers; Sanchez-Burks et al., 2016).

Considering these consequences of EA, it is highly relevant to understand why some individuals may be better at recognizing collective emotion expressions than others. Research has only started to focus on EA’s antecedents, however. While initial results have linked this ability with an individual’s tendency toward global (rather than local) information processing (Sanchez-Burks et al., 2016), we are not aware of other empirical studies that have examined EA’s origins. Hence, our conceptual and empirical knowledge about this construct remains cursory and one-sided. To advance the emerging literature on collective emotion recognition, it is important to complement the existing studies with a distinctly antecedent-focused perspective. The present study sets out to address this issue by empirically examining key factors that may promote or diminish an individual’s EA.

We specifically focus on individuals’ age as a potential influencing factor in this regard. With increasing life expectancies and later retirement ages across most industrialized nations, scholars have identified aging populations and workforces as being among the most prevalent demographic developments in recent decades (Hedge et al., 2006; Burger et al., 2012). Moreover, theory and research have repeatedly linked a person’s age with his or her ability to correctly decipher other individuals’ emotions (for a review, see Doerwald et al., 2016). It seems important, therefore, to examine whether such age-related changes may extend beyond individual emotion recognition to shape EA as well.

Interestingly, theory and research on cognitive developments across the adult lifespan point toward considerable ambiguity about the likely shape of the age-EA linkage. On the one hand, age-related growth in “crystallized” knowledge and experiences (Baltes, 1987; Ackerman and Lohman, 2006) may improve individuals’ capacity for collective emotion recognition over time. On the other hand, age-related declines in “fluid” cognitive competencies (Birren and Fisher, 1995; Salthouse, 2010) and global information processing (Oken et al., 1999) may aggravate EA, particularly among older (rather than young or middle-aged) adults. Consequently, we believe a wider theoretical approach is required to provide greater clarity on the age-EA association, integrating such cognitive explanations with a motivational perspective.

Indeed, besides cognitive capabilities, research has shown that correctly identifying other individuals’ emotions requires an actor’s motivation to attend to others’ emotion expressions (Buck, 1988; Marsh and Blair, 2008). Generalizing this notion toward EA, we propose that individuals are more likely to use their cognitive potentials to decipher group emotions to the extent they are interested in others. Logically, then, the role of age for EA should critically hinge on personality characteristics that shape this motivational orientation, and we argue that an individual’s agreeableness is particularly relevant in this regard (Costa et al., 1991). As a broad, overarching trait, agreeableness subsumes several features that closely relate with one’s concern for others and their feelings, including the tendency to be altruistic, caring, prosocial, and emotionally supportive (Digman, 1990; McCrae and John, 1992), and meta-analytic evidence has illustrated positive relationships between agreeableness and individual emotion recognition (Mayer et al., 2004; Joseph and Newman, 2010).

Building on this backdrop, our overall conceptual model casts agreeableness as a key moderator for the relationship between age and EA. By empirically testing this model in an age-diverse sample of 181 individuals, we strive to advance the nascent literature on collective emotion recognition, offering new insights into the role of key individual differences as antecedent conditions that may shape an individual’s respective ability. Beyond extending our knowledge about the origins of EA, we thereby aim to provide a novel, differentiated perspective on the role of age for EA (and, potentially, for emotion recognition in general), illustrating that a full understanding of age-related changes in this regard requires careful consideration of relevant personality characteristics as motivational boundary conditions.

### Theory and Hypothesis Development

#### The Construct of Emotional Aperture

Research has long investigated the ability to correctly recognize other *individuals’* emotion expressions (e.g., Rosenthal et al., 1979; Ekman, 2003), and a broad number of studies have identified both antecedents and consequences in this regard (for a review, see Elfenbein et al., 2002). The EA construct, by contrast, has been introduced more recently and, accordingly, the respective literature is in a more nascent state (Sanchez-Burks and Huy, 2009). Building on prior research on group emotions (e.g., George, 1990; Barsade et al., 2000), EA reflects “a person’s ability to recognize the dynamic emotional composition of a collective” (Sanchez-Burks and Huy, 2009, p. 25). As such, EA extends beyond deciphering individual members’ emotion displays toward identifying the distribution and potential heterogeneity of the emotion expressions within a group as a whole.

Consequently, scholars have emphasized that EA is both conceptually and empirically distinct from individual emotion recognition (Sanchez-Burks and Huy, 2009). In fact, a strong capacity to identify individual emotions may not suffice for an accurate assessment of a group’s overall affective composition, because the fleeting nature of affective cues may make it virtually impossible to consecutively process each member’s expressions in real time and to then aggregate these cues to the group level (Sanchez-Burks et al., 2016). Rather than representing a mere extension of individual emotion recognition, EA therefore “can be understood as using a global, or holistic processing style for encoding collective affective cues” (Sanchez-Burks et al., 2016, p. 119). Moreover, whereas measures of individual emotion recognition typically capture the ability to identify discrete emotions (e.g., anger, happiness; Nowicki and Duke, 1994), EA is assessed through the capacity to detect the distribution of positive vs. negative emotion expressions in a group (Sanchez-Burks et al., 2016). Not surprisingly, then, research has shown that (a) EA is only moderately correlated with individual emotion recognition and (b) EA exhibits incremental predictive validity for important organizational outcomes, over-and-above individual emotion recognition (Sanchez-Burks et al., 2016).

#### The Ambiguous Relationship between Age and Emotional Aperture

Lifespan theorists have pointed toward two potentially countervailing mechanisms that appear relevant for explicating the relationship between age and EA, arguing that age-related patterns of growth and decline may critically shape individuals’ social cognitions and perceptions (Phillips et al., 2002, 2008). On the one hand, certain (“crystallized”) cognitive abilities may follow a growth trajectory as individuals age, for example due to accumulated knowledge and experiences (Horn and Cattell, 1967; Baltes et al., 2006). Such learning effects may benefit one’s emotional competencies (Carstensen et al., 2000; Magai, 2001). For example, individuals’ knowledge about emotions may become more accurate and differentiated as they get older (Labouvie-Vief, 2003), enabling them to deal with emotional issues in a more efficient and automatized manner that requires less conscious effort (Suzuki and Akiyama, 2013; Morgan and Scheibe, 2014). Consequently, older individuals may become more adept at correctly deciphering both individual (Dougherty et al., 1996; Suzuki et al., 2007; Sze et al., 2012) and group emotions, suggesting a potentially positive linkage between age and EA.

On the other hand, another set of (“fluid”) cognitive abilities (including perceptual speed and working memory capacity) follow an age-related decline trajectory (Horn and Cattell, 1967; Salthouse, 2010). Emotion recognition critically requires such fluid capabilities, because it hinges on the quick and accurate recognition and discrimination of audio-visual details, identification of characteristic patterns, and comparison of these patterns with prototypes stored in memory (Adolphs, 2006; Suzuki and Akiyama, 2013). Hence, from this perspective, it is logical to assume that emotion recognition will deteriorate as individuals get older. In fact, the majority of the studies on age and individual emotion recognition support this negative association (e.g., Sullivan and Ruffman, 2004; Salthouse and Davis, 2006; Ruffman et al., 2008).

More specifically, lifespan research has illustrated that the declining age trend for many fluid cognitive capacities follows a curvilinear pattern (Horn and Donaldson, 1980; Hartshorne and Germine, 2015). These capacities generally remain relatively stable (or even increase) until about middle adulthood and diminish rather steeply afterward (Myerson et al., 2003; Hedden and Gabrieli, 2004). Consequently, scholars have argued that the negative linkage between age and individual emotion recognition may follow a curvilinear pattern as well, such that this ability may slightly improve during young and middle adulthood and decrease at older age (Williams et al., 2009; Doerwald et al., 2016). We believe this argumentation directly generalizes toward EA. In fact, EA may impose even greater demands on fluid cognitive competencies, as compared with individual emotion recognition, requiring an actor to simultaneously perceive and encode *multiple* group members’ emotional cues and to quickly integrate these – potentially diverse or even contradictory – stimuli into appropriate emotion categories in real time (Sanchez-Burks and Huy, 2009). Hence, age-related deficits in fluid cognition may yield a negative, curvilinear association between age and EA.

Additionally, as noted before, EA (rather than individual emotion recognition) uniquely requires a global, holistic style of information processing to decipher the emotionality expressed in a group as a whole (Sanchez-Burks et al., 2016). Despite some contradictory findings (e.g., Roux and Ceccaldi, 2001; Georgiou-Karistianis et al., 2006), a substantial body of research has illustrated cognitive impairments among older (as compared with younger) individuals when processing global stimuli (e.g., Slavin et al., 2002; Staudinger et al., 2011; Lithfous et al., 2016). In fact, some scholars have concluded that there may be a general shift from global toward local processing precedence with increasing age (Oken et al., 1999; Lux et al., 2008). Moreover, although this literature has rarely examined curvilinear relations, a study by Schwarzer et al. (2010) points toward this possibility, illustrating the holistic processing of facial stimuli to increase from childhood to young adulthood but to decrease afterward. Hence, beyond fluid cognitive decline, age-related detriments in global information processing may further contribute to a negative, curvilinear linkage between age and EA.

Taken together, these arguments lead to contradictory conclusions about the possible relationship between age and EA: improvements in crystallized cognition may promote this ability as individuals get older, while detriments in fluid cognition and global processing may induce curvilinear decline over time. To resolve this theoretical ambiguity, we believe it is vital to consider moderating factors in the age-EA association. Whereas the above argumentation is largely cognition-based, in particular, scholars have noted that emotion recognition also hinges on the extent to which individuals are interested in others and, thus, are motivated to attend to others’ emotion expressions and to utilize their cognitive potentials in this regard (Batson and Shaw, 1991; Goetz et al., 2010). On this basis, we integrate arguments from the lifespan literature with theory and research on personality and its underlying motivations (McCrae and John, 1992; Stanley and Isaacowitz, 2015) to propose individuals’ agreeableness as a key boundary condition for the age-EA linkage.

#### The Moderating Role of Agreeableness

As a broad dimension within the Big Five personality taxonomy (John and Srivastava, 1999), agreeableness centers around individuals’ concern for others and their tendency to value harmonious social relations (Digman, 1990; McCrae and John, 1992). Highly agreeable individuals are good-natured, cooperative, warm, and trusting, and they tend to empathize with others’ feelings (Graziano et al., 2007; Robbins et al., 2010). On this basis, it seems logical to assume that persons with relatively high agreeableness are motivated to not only attend to other individuals’ emotions (and, thus, exhibit superior individual emotion recognition; Joseph and Newman, 2010), but also to grasp the overall emotionality within relevant groups, aiding them to more smoothly navigate social interactions (cf. Sanchez-Burks and Huy, 2009). As such, we anticipate agreeableness to moderate the role of individuals’ age for their EA.

Among relatively agreeable individuals, we expect strong EA levels during young and middle adulthood. Given their deep interest in others and their tendency toward empathic concern (Melchers et al., 2016), these individuals may benefit from frequent and intense experiences with collective emotions even at a relatively young age, enabling them to quickly build an extensive knowledge base about group emotionality. In addition, these individuals should be willing to devote large parts of their fluid cognitive capacities toward deciphering collective emotion expressions, in an effort to facilitate and maintain harmonious group relations (cf. McCrae and Costa, 1987). As noted before, research has shown these capacities to be particularly pronounced among young and middle-aged adults (Salthouse, 2004; Hartshorne and Germine, 2015).

Moreover, although highly agreeable persons are likely to experience declines in fluid cognition and global processing during older adulthood (Salthouse, 2010; Staudinger et al., 2011), we believe their EA is less likely to suffer from these developments than among less agreeable individuals. Importantly, agreeable persons tend to maintain a strong interest in other individuals and groups throughout their lifespan (Donnellan and Lucas, 2008), potentially enabling a continued pattern of growth in their crystallized knowledge about dealing with emotional situations and deciphering emotional cues. Such learning effects may be particularly important for EA, given the complexity, diversity, and subtlety of collective emotion expressions (Sanchez-Burks and Huy, 2009; see also Walter et al., 2013). In fact, as noted before, accurate and differentiated emotional knowledge may allow individuals to deal with emotional situations and process affective cues in a rather routinized manner that requires little cognitive resource investment (Adolphs, 2002; Scheibe and Carstensen, 2010). Hence, we anticipate that accumulating experiences with group emotions will enable highly agreeable individuals to (at least partially) compensate for age-related cognitive declines, such that their EA should remain relatively stable even during older adulthood.

Individuals with relatively low agreeableness, by contrast, have a tendency toward self-centeredness and indifference for other persons, and they tend to exhibit little concern for others’ feelings and to attach low value to social harmony (Digman, 1990; McCrae and John, 1992). Hence, we expect a pronouncedly different pattern for the age-EA relation among less rather than more agreeable persons. During young adulthood, EA may remain relatively low among less agreeable individuals. These individuals’ knowledge and experiences about collective emotion expressions should accumulate more slowly than among their more agreeable counterparts, because their lack of empathic interest is likely to limit the frequency and intensity of their exposure to emotional group situations (cf. Habashi and Graziano, 2007). Also, despite abundant fluid cognitive capacities (Salthouse, 1996) and a tendency toward global processing during younger adulthood (Oken et al., 1999; Schwarzer et al., 2010), less agreeable individuals may lack the motivation to invest these potentials for effectively reading group emotions.

Moving from young toward middle adulthood, individuals with relatively low agreeableness may exhibit a slight increase in EA. During this age period, even less agreeable persons may benefit from accumulating experiences with other individuals’ and groups’ emotion expressions, because social life in general (Carstensen et al., 2000), and group interactions in particular (George, 1996), inevitably entail many emotion-laden encounters. Hence, crystallized knowledge about relevant affective cues and collective emotionality may increase even among low-agreeableness individuals as they approach middle adulthood – although such emotional learning may remain somewhat limited due to their lack of empathy and social interest (McCown et al., 1988). At the same time, substantive declines in fluid cognition and global processing that could be detrimental for EA are unlikely to already manifest at this age (Salthouse, 2010; Schwarzer et al., 2010).

Finally, we expect a steep drop in EA among less agreeable individuals as they progress from middle toward older adulthood. As noted before, individuals generally start to experience pronounced losses in fluid cognition and global processing at this point (Oken et al., 1999; Salthouse, 2010). Due to their lack of empathic interest in others’ feelings (Digman, 1990; Côté et al., 2011), persons with lower agreeableness may find it more difficult, as compared to their high-agreeableness counterparts, to compensate for such declines through the pronounced and continued accumulation of emotional knowledge. Consequently, they should less strongly benefit from experience and learning effects about deciphering collective emotionality at higher age, and the detrimental role of cognitive declines for EA may therefore prevail to a larger extent. All in all, we therefore predict the relationship between age and EA to follow a curvilinear pattern among individuals with less agreeableness, with relatively low EA levels during younger and older adulthood and a temporary peak around middle adulthood.

Taken together, this rationale suggests a curvilinear interaction model for the age-EA linkage, with agreeableness representing a key contingency factor. Accordingly, we hypothesize:

*Hypothesis 1*: Agreeableness moderates the curvilinear relationship between age and emotional aperture (EA). For individuals with higher agreeableness, EA remains relatively pronounced irrespective of their age. For individuals with lower agreeableness, the age-EA relation exhibits an inverted U-shape, with lower EA among younger and older than among middle-aged adults.

## Materials and Methods

### Sample and Data Collection

We aimed to recruit a heterogeneous sample for the present study to ensure sufficient variability in participants’ age, personality, and EA. Working with a group of students, we approached personal and university contacts (located in Germany) via email and social media channels with the request to participate in an on-line study on interpersonal interactions in the workplace (for similar procedures, see Bledow et al., 2013; Bunderson et al., 2016). These individuals received general information about the study (without disclosing specific hypotheses), along with a link to a secured online survey platform. Beyond demographic variables (including age), this survey incorporated a performance-based test of EA (Sanchez-Burks et al., 2016) as well as self-report measures of personality. We translated all measures to German using common back-translation procedures (Brislin, 1980). Participation was voluntary and anonymity guaranteed.

Of the targeted participants (approximately 700 individuals), 350 persons opened the survey, of which 184 provided information for all relevant study variables. Three of these participants were excluded due to excessive missing data on the EA test. Therefore, our final sample comprised 181 participants. These individuals were, on average, 38 years old (*SD* = 13.77) and covered a relatively wide age range (from 18 to 72 years). Participants were employed across a variety of organizations and industries (e.g., in manufacturing, construction, insurance, trade, and health care), with a majority (64%) working in the service sector. Fifty-three percent of the participants were female, and their average tenure with their current employer was 8 years (*SD* = 8.48).

### Measures

#### Emotional Aperture (EA)

We assessed EA using Sanchez-Burks et al. (2016) performance-based instrument. This measure consists of 17 two-frame video clips, each of which displays emotional reactions within four-person groups with differing gender and ethnicity compositions (based on the Montreal Set of Facial Displays of Emotion; cf. Beaupré and Hess, 2005). Consistent with established measures of individual emotion recognition ([e.g., the Diagnostic Analysis of Non-Verbal Accuracy (DANVA); Nowicki and Duke, 1994], the present EA test takes into account the dynamic and fleeting nature of affective cues in real-life interactions (see also Ekman, 2003). Hence, the stimulus video clips are relatively short (2s each). They initially depict a group with neutral or baseline facial expressions, followed by a second frame in which some group members exhibit an emotional reaction (i.e., changing to a different, positive or negative emotion expression) while others may retain their initial expression. After each clip, participants indicate the percentage (answer options: 0, 25, 50, or 100%) of group members that have exhibited a positive or negative emotional reaction, respectively. Due to the clips’ brevity, it is virtually impossible to focus on each individual’s expressions, and participants are therefore required to gauge a quick assessment of the global emotionality expressed within the group as a whole (Navon, 1977). A participant’s overall EA score is calculated based on the accuracy of his or her responses, representing the percentage of correct answers to all 34 items (i.e., 17 video clips, each with two responses for positive and negative emotional reactions). Hence, individual EA scores can range from 0 (all responses incorrect) to 100 (all responses correct). Sanchez-Burks and colleagues provide further details on the EA measure and its administration, along with evidence for its reliability as well as discriminant and predictive validity.

#### Age

Participants indicated their age (in years), along with other demographic variables, toward the end of the survey.

#### Agreeableness

Before implementing the EA measure, we captured participants’ agreeableness with a five-item measure based on Goldberg (1999), using a five-point response scale (1 = *strongly disagree*; 5 = *strongly agree*). Sample items included, “I am interested in people” and “I feel others’ emotions.” Cronbach’s α was 0.82.

#### Control Variables

We considered extraversion and openness to experience as possible controls because (a) these personality traits entail interpersonal aspects (Batson and Shaw, 1991; Goetz et al., 2010) and (b) research has shown these traits to associate with the ability to recognize individuals’ emotions (Matsumoto et al., 2000; Elfenbein et al., 2002). Using the same five-point response scale as for agreeableness, we assessed extraversion with four items (α = 0.74; sample item: “I start conversations”) and openness to experience with five items (α = 0.78; sample item: “I am full of ideas”), based on Goldberg (1999). Further, we included gender as a possible covariate (1 = male; 2 = female) because research has shown that women generally exhibit higher individual emotion recognition ability than men (Hall and Matsumoto, 2004). Finally, we included the industry sector (1 = service; 2 = non-service) of a participant’s employing organization as a covariate to account for potential biases related to higher emotional engagement, distinct emotion norms, and more frequent interpersonal interactions in the service industry (Ashforth and Humphrey, 1993).

## Results

### Descriptive Statistics

**Table 1** presents means, standard deviations, and bivariate correlations for all study variables. As shown, we found that age is negatively related with individuals’ EA (*r* = -0.29, *p* < 0.01), whereas EA is not significantly associated with agreeableness (*r* = 0.07, *ns*) or any of the potential control variables. Individuals’ age, however, significantly correlates with industry (such that participants working in the non-service sector are older, on average, than those working in the service sector; *r* = 0.36, *p* < 0.01), and age is negatively correlated with both openness (*r* = -0.28, *p* < 0.01) and agreeableness (*r* = -0.15, *p* < 0.05). Importantly, examining our conceptual model both with and without the control variables did not alter the significance or interpretation of the findings (cf. Becker, 2005). To illustrate the unique roles of age and agreeableness for EA, we therefore report the results including the controls in the following.

**Table 1:** Means, standard deviations, and bivariate correlations.

Variables	*M*	*SD*	1	2	3	4	5	6
(1) Industry sector	1.36	0.48						
(2) Gender	1.53	0.50	-0.33ˆ**					
(3) Extraversion	3.42	0.91	-0.09	-0.00				
(4) Openness	3.82	0.68	-0.20ˆ**	-0.07	0.41ˆ**			
(5) Agreeableness	3.96	0.72	-0.16ˆ*	0.26ˆ**	0.39ˆ**	0.31ˆ**		
(6) Age	38.17	13.77	0.36ˆ**	-0.14	-0.08	-0.28ˆ**	-0.15ˆ*	
(7) Emotional aperture	62.12	12.75	-0.07	0.07	-0.05	0.05	0.07	-0.29ˆ**

*N* = 181. Industry sector: 1 = service; 2 = non-service. Gender: 1 = male, 2 = female. ^∗^*p* < 0.05, ^∗∗^*p* < 0.01.

Moreover, to further examine the specific role of agreeableness (rather than other personality traits) for the age-EA linkage, we explored extraversion and openness to experience as possible moderating variables (i.e., we repeated our hypothesis testing by replacing agreeableness with extraversion and openness, respectively). We did not expect the respective interaction terms to be significant, because neither of these alternative personality traits shares the explicit concern for others and the empathic interest that characterizes high agreeableness (Digman, 1990; McCrae and John, 1992). And, in fact, we found none of the alternative (linear or curvilinear) interaction coefficients to be statistically significant. These supplementary findings are available from the first author.

### Hypothesis Testing

**FIGURE 1:**
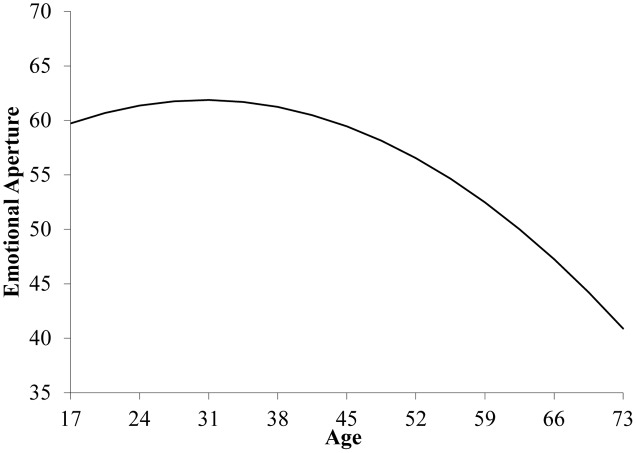
Curvilinear relationship between age and emotional aperture.

We used curvilinear moderated hierarchical regression analysis (after standardizing all continuous predictors) for hypothesis testing, entering the control variables in Step 1, the main effects for age and agreeableness in Step 2, the squared age term (age^2^) in Step 3, and the interaction terms for agreeableness with both age and age^2^ in Step 4 (Cohen et al., 2003; see **Table 2**). As shown, Step 2 of this regression analysis yields a negative linear relationship between age and EA (*B* = -3.86, *SE* = 1.03, *p* < 0.001). Moreover, as illustrated in Step 3, this association is qualified by a curvilinear pattern (*B* = -2.29, *SE* = 1.00, *p* < 0.05). Without considering the moderating role of agreeableness, EA remains relatively stable (with a slight increase) among younger individuals, with a relatively steep decline commencing after individuals’ late 30s (see **Figure 1**).

**Table 2:** Curvilinear moderated hierarchical regression analysis.

	Emotional aperture			
	Step 1	Step 2	Step 3	Step 4
Constant	61.34*** (5.10)	59.57*** (5.04)	61.24*** (5.03)	62.08*** (4.96)
**Control variables**				
Industry sector	-1.11 (2.19)	1.24 (2.20)	1.75 (2.18)	1.30 (2.15)
Gender	1.49 (2.06)	0.53 (2.08)	0.51 (2.05)	0.07 (2.02)
Extraversion	-1.11 (1.06)	-1.18 (1.08)	-1.65 (1.08)	-1.83 (1.07)
Openness	1.05 (1.09)	-0.07 (1.10)	0.09 (1.09)	-0.10 (1.09)
**Main effects**				
Agreeableness		0.80 (1.08)	0.73 (1.07)	-1.57 (1.35)
Age		-3.86*** (1.03)	-2.41* (1.20)	-2.46* (1.17)
**Squared term**				
Age^2^			-2.29* (1.00)	-1.64 (1.01)
**Interactions**				
Age × Agreeableness				-1.40 (1.17)
Age^2^ × Agreeableness				2.09** (0.78)
*R*^2^	0.02	0.09**	0.12**	0.16***
Adjusted *R^2^*	-0.00	0.05	0.08	0.10
Δ*R*^2^	0.02	0.07**	0.03*	0.04*

*Unstandardized regression weights are shown. Standard errors in parentheses.*

*^∗^*p* < 0.05, ^∗∗^*p* < 0.01, ^∗∗∗^*p* < 0.001.*

**FIGURE 2:**
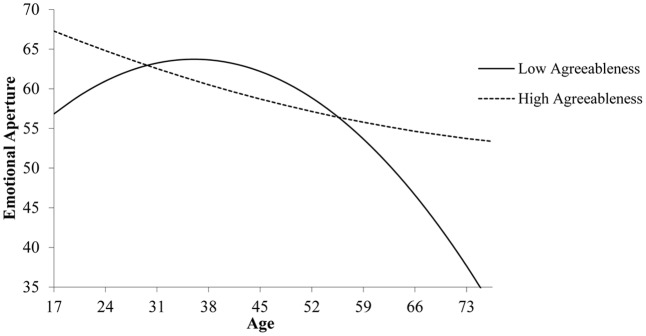
Quadratic two-way interaction of age and agreeableness on emotional aperture.

Importantly, Step 4 reveals that this curvilinear pattern does not equally apply among all individuals, with agreeableness representing a critical moderator (*B* = 2.09, *SE* = 0.78, *p* < 0.01). **Figure 2** illustrates the pattern of this curvilinear interaction. Among less agreeable individuals (-1 SD), simple slopes analyses yield an inverted U-shaped relation between age and EA (simple slope for the curvilinear term: *B* = -3.74; *SE* = 1.16, *p* < 0.01), with EA slightly increasing until these individuals’ late 30s and decreasing markedly afterward. For individuals with higher agreeableness (+1 SD), in contrast, simple slopes analyses reveal non-significant linear (*B* = -2.60; *SE* = 1.41, *p* = 0.07) and curvilinear relationships (*B* = 0.45; *SE* = 1.38, *p* = 0.75) between age and EA. As shown, these individuals exhibit relatively strong EA at younger age, with only a slight (and non-significant) declining trend over time. Hence, the present results support the curvilinear interaction model proposed in Hypothesis 1.

## Discussion

This study examined the relationship between an individual’s age and his or her ability to identify collective emotion expressions (i.e., EA). As expected, our results revealed a negative, curvilinear linkage between age and EA that was qualified by agreeableness as a key moderating factor. For individuals with higher agreeableness, EA remained relatively pronounced across the age span covered in our investigation (i.e., 18–72 years). Among less agreeable persons, by contrast, we observed a curvilinear association, such that EA remained relatively limited at both younger and older age, with a momentary peak around these individuals’ late 30s.

### Theoretical Implications

These findings make several contributions to the literature on age and (collective) emotion recognition. Scholars have only recently identified and operationalized EA as a construct that is distinct from individual emotion recognition and relates with key behavioral outcomes in organizations (Sanchez-Burks and Huy, 2009). Also, although Sanchez-Burks et al. (2016) have linked EA with individuals’ global information processing, research on EA’s antecedents has been virtually non-existent to date. Little remains known, therefore, about relevant factors that may promote or detract from an individual’s respective ability. To address this issue and widen the nomological net surrounding EA, the present study illustrates the importance of individuals’ demographic and personality characteristics as antecedent variables. By highlighting the joint roles of age and agreeableness, we promote new knowledge on why some persons may be better able than others to correctly recognize and decipher group emotions. As such, this study offers fresh insights into the origins of EA as an important, yet under-examined form of emotion recognition. Our findings seem particularly timely in the context of an aging population and an increasingly age-diverse workforce (Hedge et al., 2006; Kunze et al., 2011).

More specifically, the curvilinear interaction pattern uncovered in our research constitutes an important conceptual contribution. Integrating seemingly contradictory theoretical arguments, these findings show that one should not expect age-related changes in EA to similarly occur across all individuals. High agreeableness, in particular, may motivate individuals to closely attend to others’ emotions, enabling them to compensate for age-related cognitive losses and, thus, to retain relatively strong EA across the adult lifespan. With lower agreeableness, by contrast, a lack of empathy and interpersonal motivation may curtail individuals’ emotional experiences and limit their willingness to invest cognitive capacities for collective emotion recognition. Despite a momentary increase during younger to middle adulthood, these individuals’ EA therefore suffers markedly at higher ages. In sum, these results advance a differentiated understanding of the age-EA linkage. Focusing on individuals’ age, by itself, would provide an incomplete and inaccurate picture of EA’s development over time, with agreeableness constituting a critical contingency factor.

Finally, the curvilinear role of age among less agreeable individuals unveiled in our findings may advance the literature on general emotion recognition (i.e., beyond EA). Based on a comprehensive review of this literature, Doerwald et al. (2016, p. 166) have recently concluded that, despite rather consistent evidence for age-related deficits among older (as compared with younger) adults, “the evidence is less conclusive regarding levels of emotion perception at middle-age.” Hence, the present study provides new evidence on emotion recognition (albeit collective rather than individual) among middle-aged adults as a relatively under-studied age group. At least among individuals with relatively low agreeableness, our results corroborate Doerwald et al.’s (2016) preliminary suggestion that substantive age-related declines in emotion recognition may remain limited to older rather than middle adulthood.

### Practical Implications

The present study offers a number of suggestions for organizations aiming to improve collective emotion recognition within their workforce. Despite a trend toward age-related decline, our results show that organizations are well-advised to look beyond this demographic aspect when considering risks and potentials regarding employees’ EA. More agreeable employees, in particular, may enjoy an EA advantage largely irrespective of their age, whereas EA may be especially problematic among less agreeable employees both at relatively young and relatively old (rather than middle) age. Hence, organizations may contribute to EA among both younger and older employees by incorporating agreeableness into personnel selection procedures, fostering and communicating an agreeable organizational climate (Hofmann and Jones, 2005), and consistently emphasizing the relevance of emotional issues and emotion expressions at work toward their employees (Ashforth and Humphrey, 1995; Ashkanasy and Daus, 2002). Importantly, however, we offer these considerations with due caution. Given the limited literature on EA, we believe additional research is needed before strong practical recommendations are warranted.

### Strengths and Limitations

A key strength of the present research is its use of a validated, performance-based instrument to capture EA (Sanchez-Burks et al., 2016). Scholars have noted that such performance-based measurement is crucial for a valid assessment of emotional abilities, avoiding problems with distorted perception or socially desirable responding (Mayer et al., 2008; Joseph and Newman, 2010). Moreover, our focal measures combine direct accounts of demographic information (age) with self-reported personality assessments (agreeableness) and performance-based approaches (EA), thus ameliorating common method concerns (Spector, 2006; De Vries et al., 2014). And finally, our relatively age-diverse sample and inclusion of middle-aged participants allows for an assessment of EA’s development across a relatively long and continuous part of the adult life span, enabling us to identify curvilinear patterns that would not be discernable using extreme group designs (i.e., comparing only younger vs. older individuals) or more age-restricted samples (cf. Doerwald et al., 2016).

At the same time, this investigation has several limitations. Our theoretical reasoning has drawn on various cognitive and motivational mechanisms, for example, to explain the expected pattern of the age-EA linkage and the moderating role of agreeableness. Nevertheless, our empirical study did not incorporate these mediating factors. A relevant concern, in this regard, is that the present EA measure might overemphasize well-documented age deficits in perceptual speed (e.g., Neupert et al., 2006) due to its use of time-limited emotion stimuli (i.e., 2-s video clips; Sanchez-Burks et al., 2016). Hence, our findings might offer an inflated account of age’s negative consequences for EA, exaggerating the role of losses in fluid cognition and downplaying the role of crystallized knowledge gains. Importantly, however, scholars have noted that a time-limited measurement approach is necessary for a realistic assessment of emotion recognition, as it (a) mirrors the quick and fleeting nature of emotion expressions in real life (Nowicki and Duke, 1994; Ambadar et al., 2005; Isaacowitz and Stanley, 2011) and (b) helps to avoid possible ceiling effects (Wilhelm et al., 2014). Moreover, as outlined before, the present EA stimuli are deliberately presented so quickly that it is virtually impossible to consecutively focus on each individual’s emotion expressions, thus emphasizing global types of information processing over mere perceptual speed (Sanchez-Burks et al., 2016). And finally, we believe the moderating role of agreeableness uncovered in our study makes it unlikely that the observed age effects solely result from fluid cognitive deficits. It is clear, however, that future research could benefit from extending our model to directly assess the proposed mediating mechanisms and, thus, to assess their relative importance and further alleviate related concerns.

Moreover, like most of the previous studies on individual emotion recognition, we used a convenience sample rather than randomly selecting from a general population, and we therefore cannot rule out potential selection bias. Also, our results’ generalizability is limited due the fact that all data were collected within one country, Germany. Most previous empirical work on EA has been conducted in the United States (Sanchez-Burks et al., 2016, Studies 1 and 2 [Study 3 combined data from the United States, France, and Brazil]) and, as such, the present research extends the EA literature toward a new cultural context. At the same time, scholars have noted that cultural familiarity can benefit emotion recognition accuracy (Elfenbein et al., 2002). The present EA measure’s use of ethnically diverse stimulus groups and relatively simple (i.e., positive and negative) emotion categories may ameliorate such concerns (Sanchez-Burks et al., 2016). Nevertheless, future research that constructively replicates the present findings in alternative cultural contexts or in more diverse samples could create further confidence in our conclusions’ cross-cultural transferability.

Again mirroring the majority of the studies on age and individual emotion recognition as well as the existing research on EA (Doerwald et al., 2016; Sanchez-Burks et al., 2016), we employed a cross-sectional study design. Consequently, we cannot ascertain whether the age differences observed in the present sample arise from age or cohort effects (Rhodes, 1983), and we cannot draw strong causal conclusions. Multi-wave longitudinal designs that track multiple cohorts and repeatedly measure individuals’ EA (along with other potential antecedent variables, such as agreeableness) over extended periods of time would be helpful to address these concerns (Doerwald et al., 2016).

### Future Research Directions

Besides addressing limitations, our study offers a number of interesting directions for future research. Despite the relatively wide age range covered in our sample (i.e., 18–72 years), for instance, it is clear that our findings do not allow for conclusions about EA among younger or older individuals. With EA representing a novel and largely unexamined construct (Sanchez-Burks et al., 2016), it would therefore be worthwhile to further examine this ability within such age groups. It seems particularly interesting to investigate EA’s development through childhood and adolescence, thereby creating new knowledge on the origins of this ability during early life. Similarly, with increasing life spans in most industrialized societies (Wilmoth, 2000), it seems important to examine whether the EA decline observed in our data among less agreeable individuals continues at older ages, and whether highly agreeable individuals can maintain their relatively high EA during even later life stages.

The present study has illustrated the role of individuals’ agreeableness as a boundary condition for the age-EA linkage. Future research could extend this notion to examine contextual (rather than individual) moderators. In organizational settings, in particular, explicit or implicit display rules and emotion norms may influence how employees express and perceive emotions (Rafaeli and Sutton, 1989; Diefendorff and Richard, 2003). Such rules and norms might shape EA among employees that spend large parts of their working lives within the respective organization, potentially altering age-related developments in this capacity. Similarly, research has demonstrated that perceived closeness between an actor and a target may attenuate the negative linkage frequently observed between age and individual emotion recognition (Zhang et al., 2013). It seems worthwhile, therefore, to examine whether the age-EA linkages uncovered in our research would similarly occur among participants that feel a closer psychological bond with the target group (e.g., due to strong social identification). Furthermore, prior studies have shown that older individuals tend to pay closer attention to positive rather than negative information (Isaacowitz et al., 2006; Horning et al., 2012). To better understand the age-EA association, it may be interesting to examine how this positivity bias (Mather and Carstensen, 2005) might influence older individuals’ EA and shape the moderating role of agreeableness in this regard.

Moreover, as noted before, individuals working in the non-service sector were older and less agreeable than individuals working in the service sector in the present study (see **Table 1**). Hence, although industry sector and EA were not significantly related (and although controlling for industry sector did not change the pattern and significance of our findings), it would be interesting to further explore whether the curvilinear interaction of age and agreeableness on EA might differ across industries. The present sample’s size is too limited to address this question with confidence. Hence, future research could benefit from addressing this issue in larger samples with more balanced industry representation.

Finally, beyond addressing concerns about causality and cohort effects, longitudinal studies that capture both EA and agreeableness at multiple points in time could enable an interesting extension of the present considerations, modeling age-related EA changes in parallel with potential age-related personality developments. While we have found a negative relationship between age and agreeableness (*r* = -0.15, *p* < 0.05; see **Table 1**), prior research on this issue has produced somewhat mixed results (for an overview, see Specht, 2017). Meta-analytic evidence, for example, points toward relative stability in agreeableness during young and middle adulthood and a slight increase among older adults (Roberts et al., 2006; see also Roberts and Mroczek, 2008), whereas some recent studies have reported stable (e.g., Kandler and Bleidorn, 2015) or even decreasing (e.g., Wortman et al., 2012) agreeableness at older ages. Hence, longitudinal research could enable a more dynamic perspective on how age-related agreeableness trajectories could shape the age-EA linkage over time.

## Conclusion

The present research illustrates that individuals’ EA jointly hinges on the complex interplay of their age and agreeableness. As such, this study offers relevant insights into the antecedents of EA as an important, yet rarely examined construct. We hope our findings will stimulate additional research efforts related to the predictors and consequences of collective emotion recognition, hence offering a tangible contribution to the empirical knowledge base on individuals’ emotional abilities.

## Ethics Statement

As an online study with voluntary and anonymous participation, no specific manipulations, and no deception, the authors’ university did not require ethics approval for this research.

## Author Contributions

Both authors provided ideas, planned the study, and edited the manuscript. AF collected the data, conducted analyses, and wrote initial drafts of the manuscript.

## Conflict of Interest Statement

The authors declare that the research was conducted in the absence of any commercial or financial relationships that could be construed as a potential conflict of interest.
